# Microbial survey of ready-to-eat salad ingredients sold at retail reveals the occurrence and the persistence of *Listeria monocytogenes* Sequence Types 2 and 87 in pre-packed smoked salmon

**DOI:** 10.1186/s12866-017-0956-z

**Published:** 2017-02-28

**Authors:** Man Ling Chau, Kyaw Thu Aung, Hapuarachchige Chanditha Hapuarachchi, Pei Sze Valarie Lee, Pei Ying Lim, Joanne Su Lin Kang, Youming Ng, Hooi Ming Yap, Hyun-Gyun Yuk, Ramona Alikiiteaga Gutiérrez, Lee Ching Ng

**Affiliations:** 1 Environmental Health Institute, National Environment Agency, 11 Biopolis Way, #04-03/04, Helios Block, Singapore, 138667 Singapore; 2 Department of Chemistry, Food Science and Technology Programme, National University of Singapore, 3 Science Drive 3, Singapore, 117543 Singapore; 3 School of Biological Sciences, Nanyang Technological University, 60 Nanyang Drive, Singapore, 637551 Singapore

**Keywords:** Microbial safety and quality, Salads, Smoked salmon, *Listeria monocytogenes*, Multi Locus Sequence Typing (MLST), Food safety

## Abstract

**Background:**

As the preparation of salads involves extensive handling and the use of uncooked ingredients, they are particularly vulnerable to microbial contamination. This study aimed to determine the microbial safety and quality of pre-packed salads and salad bar ingredients sold in Singapore, so as to identify public health risks that could arise from consuming salads and to determine areas for improvement in the management of food safety.

**Results:**

The most frequently encountered organism in pre-packed salad samples was *B. cereus,* particularly in pasta salads (33.3%, 10/30). The most commonly detected organism in salad bar ingredients was *L. monocytogenes,* in particular seafood ingredients (44.1%, 15/34), largely due to contaminated smoked salmon. Further investigation showed that 21.6% (37/171) of the pre-packed smoked salmon sold in supermarkets contained *L. monocytogenes*. Significantly higher prevalence of *L. monocytogenes* and higher Standard Plate Count were detected in smoked salmon at salad bars compared to pre-packed smoked salmon in supermarkets, which suggested multiplication of the organism as the products move down the supply chain. Further molecular analysis revealed that *L. monocytogenes* Sequence Type (ST) 2 and ST87 were present in a particular brand of pre-packed salmon products over a 4-year period, implying a potential persistent contamination problem at the manufacturing level.

**Conclusions:**

Our findings highlighted a need to improve manufacturing and retail hygiene processes as well as to educate vulnerable populations to avoid consuming food prone to *L. monocytogenes* contamination.

**Electronic supplementary material:**

The online version of this article (doi:10.1186/s12866-017-0956-z) contains supplementary material, which is available to authorized users.

## Background

Salad dishes are cold ready-to-eat (RTE) dishes that typically contain raw cuts of vegetables as well as other cooked and smoked ingredients. As salads contain large portions of raw ingredients and their preparation involves extensive handling processes, they are exposed to a higher risk of microbial contamination than other ready-to-eat dishes which are mostly cooked. Although no major outbreaks associated with salads have been reported in Singapore, there are numerous reports on outbreaks worldwide associated with salad vegetables and salad dishes contaminated with pathogens such as *Listeria monocytogenes, Shigella sonnei, Salmonella* spp. and *Escherichia coli* O157: H7 [1–5]. Ingredients used in salad dishes can be contaminated through various routes along the supply chain. For instance, fresh produce can be contaminated through dirty irrigation water, soil or poor hygiene practices during harvesting [1]. At the processing plants, the use of unclean water for washing, in chill tanks or as shipping ice can also result in the contamination of fresh produce [1]. At the retail level, cross-contamination and bacterial growth can occur due to improper segregation of utensils for handling raw and cooked ingredients, poor hygiene practices or inadequate chilling [1]. Thus, it is of public health interest to assess the microbial safety and quality of RTE salad dishes sold at retail.

According to Singapore’s national surveillance statistics [6, 7], the annual incidence of polyclinic attendances due to acute diarrhoea was about 2.4% of the population in 2011 and 2012; the annual incidence of food poisoning outbreaks (two or more notified cases epidemiologically linked to a common source) were 4.7 and 5 per 100,000 populations respectively [6, 7]. During outbreak investigations between 2009 and 2011, *S. aureus* was identified to be the most common foodborne organism detected in food samples; *Salmonella* spp. was the most frequently encountered pathogen in stool samples of cases [8]*.* While cases of campylobacteriosis, cholera, hepatitis A, hepatitis E, paratyphoid, typhoid and salmonellosis are legally notified by medical doctors and diagnostic laboratories, symptom-based notifications are also received from the medical community and from the public through various channels such as government hotlines and emails [8]. Among the notifiable diseases, non-typhoidal salmonellosis and campylobacteriosis have shown an increasing incidence in Singapore over the years [6, 7, 9–11].

In this study, we aimed to assess the microbial safety and quality of retail salads sold in Singapore through the sampling of pre-packed salads and salad bar ingredients. *L. monocytogenes, Salmonella* spp., and *E. coli* O157:H7 were screened for in samples collected in this study as these pathogens were previously detected in salad vegetables or salad dishes in other countries [1–5]. Poultry-containing samples were screened additionally for the presence of *Campylobacter* spp. as human campylobacteriosis is largely linked to undercooked or improperly handled poultry items [12]. Similarly, seafood-containing samples were screened for the presence of *V. cholerae* and *V. parahaemolyticus* as human infections caused by these pathogens are mostly associated with the consumption of raw or undercooked seafood items [12]. Hygiene indicators including non-pathogenic *E. coli, S. aureus* and *B. cereus* were also tested for to make inference on the occurrence of hygiene lapses during food preparation. While non-pathogenic *E. coli* is generally used as an indicator for undercooking, faecal and/or post-cooking contamination; *S. aureus* is used as an indicator for poor hand hygiene; *B. cereus* as an indicator for time-temperature abuse during storage [13].

Due to the detection of a high prevalence of *L. monocytogenes* in smoked salmon from salad bars, the microbial safety and quality of pre-packed smoked salmon distributed at local supermarkets was also investigated. In addition, smoked salmon products of a particular brand were collected annually for another 3 years to monitor the presence of persistent *L. monocytogenes* strains. Findings gathered from this study are important to identify potential health risks that could arise from consuming salads and to determine measures for improving retail food hygiene and safety. To our knowledge, this is the first report on the microbial safety and quality of RTE pre-packed salad dishes and salad bar ingredients sold in Singapore.

## Methods

### Collection of food samples

There were three phases of sampling in this study. Phase I was a survey on the microbial safety and quality of pre-packed salads and salad bar ingredients sold at retail food establishments. Phase II was a survey on the microbial safety and quality of pre-packed smoked salmon sold at supermarkets. Phase III was an extension of Phase II sampling of a brand of products (Brand A) which was shown to be contaminated by *L. monocytogenes* ST2 and ST87 in multiple batches of pre-packed smoked salmon over a 1-year period. Phase III served to gather preliminary evidence to prompt further investigation upstream to address a suspected contamination problem associated with the repeated detection of ST2 and ST87 in Brand A products over another 3-year period.

Phase I comprised the sampling of 106 pre-packed salads and 198 salad bar ingredients across Singapore between September 2011 and January 2012. Pre-packed salads, which included vegetable salads (*n* = 44), chicken salads (*n* = 32) as well as pasta, rice and couscous salads (*n* = 30), were purchased from 54 retail food shops. Thirty-seven packets of dressing sold with the pre-packed salad samples were analysed separately. Six types of salad bar ingredients, which included seafood (*n* = 34), dressing (*n* = 34), pasta, rice and couscous (*n* = 34), vegetable (*n* = 33), poultry and eggs (*n* = 32) and cheese (*n* = 31) samples were purchased from 24 salad bars. Premises were identified through crowd sourcing websites on local retail food and beverage businesses that were known to be salad bars or known to sell RTE pre-packed salad dishes. A minimum sample size of 30 was targeted for each type of pre-packed salad or salad bar ingredient for statistical comparison. This is to achieve a level of confidence of more than 80%, with the assumptions that the population is infinite and the prevalence of each foodborne pathogen is 50% (worst case scenarios). The number of samples collected was spread across the various parts of Singapore; products of popular brands and those commonly available to the public were sampled. Descriptions of ingredients in pre-packed salads and the breakdown of salad bar ingredients collected in this study were provided in Additional file 1: Table S1 and S2 respectively.

Phase II consisted of the sampling of 171 pre-packed smoked salmon samples between December 2011 and September 2012. This is to achieve a level of confidence of more than 95%, with the assumptions that the population is infinite and the prevalence of *L. monocytogenes* is about 87% - the prevalence of *L. monocytogenes* observed in smoked salmon at salad bars. Seventeen brands (representing 17 manufacturers) of smoked salmon samples were purchased from 19 supermarkets. At each supermarket, one sample was taken from each brand, each product variety (flavour/packaging) and each batch which was available during the time of sampling. Thus, more samples were collected from brands with wider varieties of flavoured smoked salmon products, as well as from those with multiple batches on display. Based on information declared on the packaging of pre-packed smoked salmon, the countries of origin of salmon were Australia, Chile, Denmark, Ireland, Korea, New Zealand, Norway and Scotland; the countries of salmon processing were Australia, Denmark, France, Korea, Malaysia, New Zealand, Norway, Scotland, Singapore, Sweden and the Philippines.

Phase III consisted of the sampling of pre-packed salmon sashimi samples (*n* = 2) in 2013, pre-packed smoked salmon samples (*n* = 8) in 2014 and pre-packed salmon sashimi sample (*n* = 1) in 2015 from Brand A. These 11 samples were analysed for the presence of *L. monocytogenes* and persistent sequence types detected in Phase II. The sample size was small as the intention was to provide some preliminary evidence to prompt for further investigation at the food manufacturing site.

All pre-packed samples were purchased in their original packaging, whereas the individual salad bar ingredients were collected in sterile bags. All samples were transported on ice, kept refrigerated in the laboratory and analysed within 24 h from the time of purchase.

### Microbiological analyses

#### Microbiological analyses involving the use of pre-enriched sample aliquots

Ten grams of each sample were homogenised with 90 ml of Universal Pre-enrichment Broth (UPB) using a Stomacher (model 400, Seward Medical, England) with a paddle speed of 230 rpm for 30 s. The pre-enriched aliquots were used for the determination of Standard Plate Count (SPC), *E. coli* and *S. aureus* counts according to methods previously described [14]. Additional tests were performed to determine *Bacillus cereus* and *L. monocytogenes* counts as well as the presence of extended spectrum β-lactamase producing *E. coli* strains as outlined below.

(I) *Bacillus cereus* count

One hundred microlitres of each serial diluted pre-enriched aliquot (from neat to 10^4^ dilutions) were plated onto Mannitol Egg Yolk Polymyxin agar (Oxoid) and incubated at 30 °C for 24 h. Presumptive pink colonies with opaque zones were confirmed by observing the absence of crystal toxins in bacterial stains using a published method [15], which involved the use of 0.133% Coomassie Blue dye (Merck, Darmstadt, Germany) in 50% acetic acid (Merck).

(II) *Listeria monocytogenes* count

One millilitre of each serial diluted pre-enriched aliquot (from neat to 10^6^ dilutions) was plated onto PALCAM agar (Oxoid) and incubated at 37 °C for 48 h. Presumptive grey-green colonies surrounded by dark halos on PALCAM agar were picked for further confirmation by morphology using chromID^TM^ Ottaviani Agosti agar (bioMérieux, France) and biochemically using RAPiDEC® Lmono (bioMérieux) following the manufacturer’s instructions. The level of contamination was quantified only for those collected during the latter part of this study upon noticing an unexpectedly high prevalence of *L. monocytogenes* in smoked salmon samples. Those samples included seven of the positive smoked salmon samples collected from salad bars (*n* = 13), and 34 positive pre-packed smoked salmon samples obtained from supermarkets (*n* = 37).

(III) Extended spectrum β-lactamase producing *Escherichia coli* strains

Upon the quantification of *E. coli* count using a previously described method involving pre-enriched aliquots [14], the isolates were streaked onto Brilliance ESBL agar (Oxoid, Hants, UK) and incubated at 37 °C for 24 h for the detection of extended spectrum β-lactamase producing strains.

#### Microbiological analyses using post-enriched sample aliquots

The remaining UPB aliquots were incubated and enriched at 37 °C for 24 h for the detection of *E. coli* O157: H7, *L. monocytogenes* and *Salmonella* spp. using previously described methods [14] with additional tests performed to detect for the presence of *V. cholerae and V. parahaemolyticus* outlined below. The effective use of UPB for simultaneous enrichment of pathogens was demonstrated in several studies [16–18]; the evaluation of UPB as a suitable sample suspension-and-enrichment medium for this study was described in Additional file 2: Appendix S1 and Figure S1. A portion of the food sample was also kept separately for the testing of *Campylobacter* spp. using method as follows.

(I) *Vibrio cholerae* and *Vibrio parahaemolyticus*

The screening of *V. cholerae* and *V. parahaemolyticus* was conducted only on pre-packed seafood salad dishes, seafood salad bar ingredients and pre-packed smoked salmon using the post-enriched aliquots. A 10 μl-loopful of the post-enriched culture in UPB was sub-cultured onto Thiosulfate-Citrate-Bile Salt-Sucrose (TCBS) agar (Acumedia) for the isolation of *V. cholerae* and *V. parahaemolyticus*. Presumptive large green and yellow colonies with on TCBS were further analysed using CHROMagar^TM^ *Vibrio* (Oxoid), API 20 E (bioMérieux), oxidase (Oxoid) for the confirmation of *V. parahaemolyticus* and *V. cholerae* respectively according to manufacturers’ instructions.

(II) *Listeria monocytogenes*

A 10 μl-loopful of the post-enriched culture in UPB was sub-cultured onto PALCAM agar for the isolation of *L. monocytogenes.* Presumptive *L. monocytogenes* colonies on PALCAM were verified using confirmatory methods similar to the steps mentioned in the above spread plate method.

(III) *Campylobacter* spp

For pre-packed chicken salads, as well as poultry and egg ingredients from salad bars, 10 g of each food sample were homogenised with 90 ml of Bolton Selective Enrichment Broth (Oxoid, Hamshire, UK) and incubated at 42 °C for 48 h for the detection of *Campylobacter* spp.. A 10 μl-loopful of the post-enriched culture in Bolton broth was streaked onto modified Charcoal-Cefoperazone-Desoxycholate agar (mCCDA) (Oxoid) and incubated at 42 °C for 48 h under microaerophilic conditions. Presumptive grey colonies on mCCDA were isolated for further confirmation using oxidase test, serological latex agglutination (Remel) and API Campy (bioMérieux) following manufacturers’ instructions. Microaerophilic conditions were maintained using Campygen sachets (Oxoid) in sealed jars.

### Detection of bacteria and their virulence genes by polymerase chain reaction

*Escherichia coli* isolates were screened for the presence of virulence genes associated with human diarrhoeal diseases as described previously [19–21] (see Additional file 3: Table S3). Amplification was performed in a thermal cycler (ABI Systems GeneAmp PCR system 9700) with the following temperature ramping: 98 °C for 30 s, followed by 35 cycles of 98 °C for 10 s, 64 °C for 30 s, 72 °C for 30 s, and finally 72 °C for 10 min. Amplified products were analysed using QIAxcel DNA High Resolution Kit (Qiagen, Hilden).

The presence of methicillin-resistant gene (*mecA*) and enterotoxin-producing genes (*SEA, SEB, SEC, SED, SEE, SEG, SEH, SEI, SEJ, SEL*) of *S. aureus* was determined as described before [22–24] (see Additional file 3: Table S3). Amplification was performed with the following temperature ramping: 98 °C for 30 s, followed by 30 cycles of 98 °C for 10 s, 61 °C for 30 s, 72 °C for 30 s, and finally 72 °C for 10 min. Amplified products were analysed using 2% agarose gel electrophoresis.

The presence of emetic toxin gene (*cer*), diarrhoeal toxin genes (*hblCDA, nheABC, cytK, entFM, bceT*) and haemolysin II gene (*hlyll)* of *Bacillus* isolates was determined as described previously [25–32] (see Additional file 3: Table S3). Amplification was performed with the following temperature ramping: 98 °C for 30 s, followed by 35 cycles of 98 °C for 10 s, 60 °C for 30 s, 72 °C for 30 s, and finally 72 °C for 10 min. Amplified products were analysed using QIAxcel DNA High Resolution Kit (Qiagen, Hilden).

*L. monocytogenes* isolates were confirmed with a *Listeria* genus-specific PCR and a *L. monocytogenes* species*-*specific PCR which targeted the *prs* gene [33] and *inlA* gene [34] (see Additional file 3: Table S3). Amplification was performed with the following temperature ramping: 98 °C for 30 s, followed by 30 cycles of 98 °C for 10 s, 61 °C for 30 s, 72 °C for 30 s, and finally 72 °C for 10 min. Amplified products were analysed using 2% agarose gel electrophoresis.

All bacterial DNA was extracted using QIAamp DNA Mini Kit (Qiagen, Hilden) according to the manufacturer’s instructions.

### Characterisation of *L. monocytogenes*

(I) Multi Locus Sequencing Typing (MLST)

Sixty-four *L. monocytogenes* isolates obtained in Phase I and II studies, as well as eight isolates obtained from a brand of salmon products in Phase III study were characterised using the MLST method. The method involved the amplification of seven housekeeping gene regions, namely *abcZ, bglA, cat, dapE, dat, ldh* and *lhkA* using published primers and protocol [35] (see Additional file 3: Table S3). Amplified products were sequenced using the BigDye Terminator Cycle Sequencing Kit (Applied Biosystems, USA). Nucleotide sequences obtained were then aligned using Seqman Pro Software version 8.0 (DNASTAR, USA) and compared with sequences available in the Institut Pasteur *Listeria monocytogenes* MLST database (http://bigsdb.pasteur.fr/listeria/), so as to obtain information on the allelic profiles and Sequence Type (ST) of each isolate. A phylogenetic tree was constructed using the Molecular Evolutionary Genetics Analysis (Mega) software version 7.0 [36]; using the Neighbour-Joining (NJ) method based on concatenated nucleotide sequences of seven MLST house-keeping genes of sequence types detected in Phase I and II studies.

(II) Serotyping

Serotyping was carried out using the *Listeria* antisera set (Denka Seiken Co., Ltd, Japan), following the manufacturer’s instructions.

### Statistical analysis

As microbial data were not normally distributed, statistical differences (*p* < 0.05, *p* < 0.005 and *p* < 0.001) in SPC levels (log CFU/g) among various types of pre-packed salad dishes and salad bar ingredients were evaluated respectively using a non-parametric test, specifically the Kruskal-Wallis Test. Multiple pairwise comparison of SPC levels (log CFU/g) between any two types of pre-packed salad dishes or salad bar ingredients were evaluated using a non-parametric test, specifically the Mann-Whitney Test. Statistical analysis was performed using SPSS v22.0 software.

## Results

### Microbial analyses of salad bar ingredients and pre-packed salads

**Fig. 1:**
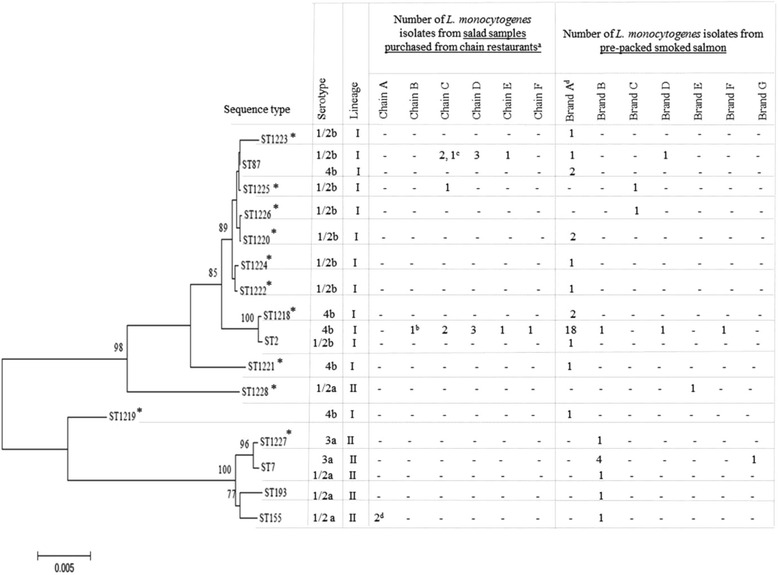
Phylogenetic tree using the Neighbour-Joining (NJ) method based on concatenated nucleotide sequences of seven MLST house-keeping genes of 16 Sequence Types (ST) of 64 *L. monocytogenes* isolates recovered from salad and smoked salmon samples collected between September 2011 and October 2012 (phases I & II). Bootstrap values of >70% are shown on the branches and are calculated from 1,000 replicates. The horizontal scale bar of 0.005 represents nucleotide substitution rate per site. ^*^Novel sequence types determined in this study (http://bigsdb.pasteur.fr/listeria/) ^a^ Isolates were recovered from smoked salmon samples unless specified. Isolates were recovered from ^b^a chilled cooked prawn sample, ^c^a chilled cooked shrimp sample and ^d^pre-packed chicken salad samples. ^d^A total of 31 isolates were recovered from 24 samples of Brand A’s pre-packed smoked salmon. Six samples were found positive with two different *L. monocytogenes* STs. These include the co-detection of ST2 and ST1218 in two samples; the co-detection of ST2 and ST1220, ST2 and ST1221, ST1220 and ST1222, as well as ST87 and ST1223 in one sample respectively

Table 1 shows the percentages of samples positive for specific foodborne bacteria and the range of contamination levels. The most frequently encountered organism in salad bar ingredients was *L. monocytogenes,* particularly in seafood ingredients (44.1%, 15/34). Smoked salmon contributed to 86.7% (13/15) of the seafood ingredients that were positive for *L. monocytogenes*. A total of 18 *L. monocytogenes* colonies obtained from the sampling of pre-packed salads and salad ingredients was characterised using serotyping and Multi Locus Sequence Typing (MLST) methods (see Fig. 1). The most common sequence types determined were ST 2 (serotype 4b) and ST87 (serotype 1/2b). It was also interesting to note the presence of ST155 in pre-packed chicken salads obtained from 2 geographically distant outlets of a salad bar chain (Chain A) and the presence of ST87 in one smoked salmon sample and one chilled cook shrimp sample collected from an outlet of a salad bar chain (Chain C).

**Table 1:** Prevalence of foodborne bacteria in pre-packed salads, salad bar ingredients and smoked salmon samples collected between September 2011 and September 2012 (phases I and II)

	Percentage (%) of positive samples (Range of bacterial counts detected in positive samples, log CFU/g)			
	*L. monocytogenes*	*B. cereus*	*E.coli*	*S. aureus*
Salad bar ingredients				
Seafood	44.1% (15/34)	2.9% (1/34)	2.9% (1/34)	0% (0/34)
		(2.0 log CFU/g)	(3.4 log CFU/g)	
Poultry & eggs	0% (0/32)	6.3% (2/32)	6.3% (2/32)	3.1% (1/32)
		(2.7 log CFU/g)	(1.6 – 1.8 log CFU/g)	(1.9 log CFU/g)
Dressing	0% (0/34)	2.9% (1/34)	0% (0/34)	0% (0/34)
		(2.3 log CFU/g)		
Pasta, rice & couscous	0% (0/34)	0% (0/34)	5.9% (2/34)	0% (0/34)
			(2.0 – 2.5 CFU/g)	
Vegetable	0% (0/33)	0% (0/33)	0% (0/33)	6.1% (2/33)
				(3.2 log CFU/g)
Cheese	0% (0/31)	0% (0/31)	0% (0/31)	0% (0/31)
Pre-packed salads				
Pasta, rice& couscous salads	0% (0/30)	33.3% (10/30)	0% (0/30)	0% (0/30)
		(2.0 – 2.7 log CFU/g)		
Vegetable salads	0% (0/44)	15.9% (7/44)	2.3% (1/44)	0% (0/44)
		(2.0 – 3.6 log CFU/g)	(1.7 log CFU/g)	
Chicken salads	6.3% (2/32)	3.1% (1/32)	3.1% (1/32)	0% (0/32)
		(2.0 log CFU/g)	(1.0 log CFU/g)	
Pre-packed dressing	0% (0/37)	0% (0/37)	0% (0/37)	0% (0/37)
Smoked salmon				
Unsealed items displayed at salad bars^a^	86.7% (13/15)	6.7% (1/15)	0% (0/15)	0% (0/15)
	(<1.0 – 4.9 log CFU/g)^b^	(2.5 log CFU/g)		
Pre-packed items at Supermarkets	21.6% (37/171)	1.8% (3/171)	0% (0/171)	0% (0/171)
	(<1.0 – 5.4 log CFU/g)^c^	(2.5 – 3.4 log CFU/g)		

^a^ A subset of seafood ingredients sold at salad bars

^b^ *L. monocytogenes* counts were determined in 7 of the 13 positive samples, median count = 3.5 log CFU/g

^c^ *L. monocytogenes* counts were determined in 34 of the 37 positive samples, median count = 3.1 log CFU/g

The most frequently encountered organism in pre-packed salad samples was *B. cereus,* particularly in pasta, rice and couscous salads (33.3%, 10/30) (see Table 1). An overall higher prevalence of *B. cereus* was observed in pre-packed salad samples (12.6%, 18/143) compared to salad bar ingredients (2.0%, 4/198). A total of 33 *B. cereus* colonies obtained from the sampling of pre-packed salads and salad ingredients was screened for the presence of virulence genes. Three isolates possessed five diarrhoeal enterotoxin genes (*hblCDA, nheABC, cytK, entFM, bceT*) and the haemolysin II gene (*hlyII*). The emetic toxin gene (*ces*) was detected in three other *B. cereus* isolates and 13 isolates possessed at least three diarrhoeal enterotoxin genes. Although virulence genes were detected in several *B. cereus* strains isolated in this study, the counts (2.0 – 3.6 log CFU/g) were lower than the levels (5 – 8 log CFU/g) that are indicative of potential human hazard due to preformed toxins [12].

*Escherichia coli* was occasionally detected in pre-packed salads (1.4%, 2/143) and salad bar ingredients (2.5%, 5/198) (see Table 1). Most *E. coli* positive samples (85.7%, 6/7) were contaminated with levels exceeding the local regulatory limit of 1.3 log CFU/g in ready-to-eat food [37]. All *E. coli* isolates obtained in this study were extended spectrum beta lactamase (ESBL)–negative and were not found to possess virulence genes or groups of virulence genes known to be associated with diarrhoeal diseases in humans.

*Staphylococcus aureus* was not detected in any pre-packed salad samples tested but was occasionally detected in vegetable ingredients (6.1%, 2/33), as well as poultry and egg ingredients (3.1%, 1/32) from salad bars (see Table 1). All *S. aureus* isolates in this study were methicillin-sensitive and none was found to possess enterotoxin genes (*SEA, SEB, SEC, SED, SEE, SEG, SEH, SEI, SEJ* and *SEL*). All counts were lower than the level that can produce sufficient enterotoxins to cause food poisoning (5 log CFU/g) [12].

**Fig. 2:**
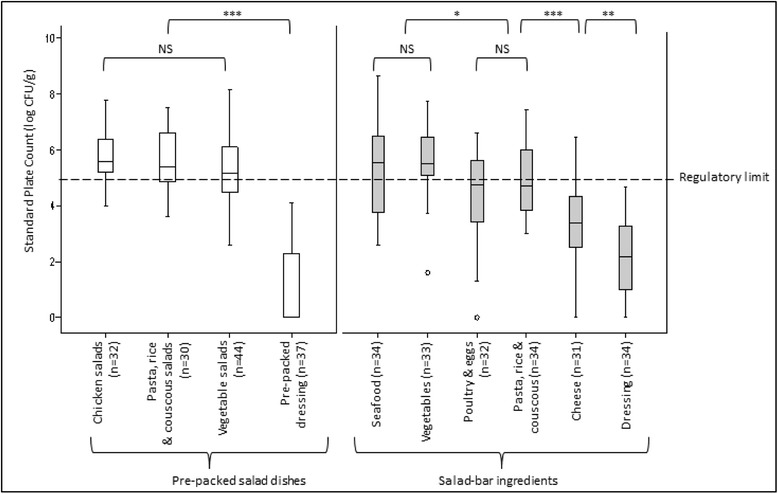
Standard Plate Count (SPC) of pre-packed salads and salad bar ingredients. NS: The difference in SPC was not significant (*p* > 0.05). *: The difference in SPC was significant (*p* < 0.05). **: The difference in SPC was significant (*p* < 0.005). ***: The difference in SPC was significant (*p* < 0.001). Outliers. ---: Singapore’s regulatory limit for SPC in ready-to-eat food (<5 log CFU/g) [37]

No significant difference was observed in SPC among the various types of pre-packed salads (see Fig. 2). The median SPC of chicken salads, pasta, rice and couscous salads as well as vegetable salads were 5.6 log CFU/g, 5.4 CFU/g and 5.2 log CFU/g respectively. The median SPC of pre-packed dressing was <1 log CFU/g and the counts were significantly lower (*p* < 0.001) than those of pre-packed salad samples.

Among the various salad bar ingredients (see Fig. 2), seafood (5.6 log CFU/g) and vegetable ingredients (5.5 log CFU/g) showed the highest median SPC. The median SPC of poultry and egg ingredients, as well as pasta, rice and couscous ingredients were 4.8 log CFU/g and 4.7 log CFU/g respectively, and the counts were significantly lower (*p* < 0.05) than those of seafood and vegetable ingredients. The lowest median SPC was observed in cheese (3.4 log CFU/g) and dressing ingredients (2.2 log CFU/g).

*Escherichia coli* O157: H7 and *Salmonella* spp. were not detected in any samples. Only pre-packed chicken salads, as well as poultry and egg ingredients from salad bars were tested for *Campylobacter* spp. and all results were negative. Likewise, only pre-packed seafood salads and seafood ingredients from salad bars were tested for *V. cholerae* and *V. parahaemolyticus*, and all results were negative.

### Microbial analyses of pre-packed smoked salmon

Due to a high contamination rate of *L. monocytogenes* observed in smoked salmon from salad bars, a follow up study was conducted to assess the microbial safety and quality of pre-packed smoked salmon sold at supermarkets between December 2011 and September 2012. Our results show that the prevalence of *L. monocytogenes* in pre-packed smoked salmon from supermarkets (21.6%, 37/171) was significantly lower (*p* < 0.001) than smoked salmon displayed at salad bars (86.7%, 13/15) (see Table 1). Similarly, significantly lower SPC (*p* < 0.001) was detected in pre-packed smoked salmon from supermarkets compared to smoked salmon from salad bars (see Additional file 4: Figure S2). The median SPC of pre-packed salmon from supermarkets was 4.6 log CFU/g and the median SPC of smoked salmon from salad bars was 6.5 log CFU/g.

Further molecular characterisation of *L. monocytogenes* isolates recovered from pre-packed smoked salmon revealed five known sequence types and 11 novel sequence types (http://bigsdb.pasteur.fr/listeria/) (see Fig. 1). While ST2 and ST87 were detected repeatedly in 19 and 3 samples, respectively) in one particular brand of pre-packed smoked salmon (Brand A); ST7 was detected repeatedly (in 5 samples) in another brand (Brand B). Among the 17 brands of pre-packed smoked salmon sampled (each brand representing a manufacturer), the prevalence of *L. monocytogenes* was exceptionally high (80%, 24/30) in Brand A products. Further investigation was performed by testing Brand A’s pre-packed salmon products annually for another 3 years, which detected another seven *L. monocytogenes* ST2 strains from a salmon sashimi sample collected in 2013, five smoked salmon samples collected in 2014 and a salmon sashimi sample collected in 2015. One *L. monocytogenes* ST87 strain was also isolated from a salmon sashimi sample collected in 2013.

The sampling of pre-packed smoked salmon from supermarkets between September 2011 and January 2012 also detected three *B. cereus* –positive samples (Table 1). An isolate from one of these samples was found to possess two diarrhoeal enterotoxin genes, namely *nheABC* and *entFM*. All *B. cereus* counts detected were much lower than the levels (5 – 8 log CFU/g) that are indicative of potential human hazard due to preformed toxins [12]. *Escherchia coli* and *S. aureus* counts, as well as *E. coli* O157: H7, *Salmonella* spp., *V. cholerae* and *V. parahaemolyticus* were not detected.

## Discussion

### The detection of *L. monocytogenes* in smoked salmon and salads highlighted a need to improve manufacturing and retail hygiene processes

Among various foodborne bacteria, *L. monocytogenes* is often highlighted as a food safety concern in RTE food [38–41] as the organism is ubiquitous in the environment and can grow at refrigerated temperatures [12]. Though *L. monocytogenes* generally causes mild or no symptoms in healthy individuals, it can result in life threatening consequences among the vulnerable populations, particularly pregnant women who may suffer from miscarriages or stillbirths [12]. The *L. monocytogenes* contamination rate of pre-packed smoked salmon collected from supermarkets (21.6%, 37/171) in our study was comparable to other countries where the organism was detected in 16% to 32% of the retail smoked salmon tested [40, 42, 43]. In the present study, the contamination was largely due to a single brand (Brand A). The recurrence of *L. monocytogenes* ST2 strains and ST87 strains in multiple batches of Brand A’s salmon products over a 4-year period suggested a potential persistent contamination issue at the salmon processing plant. Such persistency has been demonstrated in previous studies using random amplified polymorphic DNA (RAPD), and biofilms on difficult-to-clean food contact surfaces or machinery parts in salmon smoking plants were postulated as the source of contamination [44, 45]. Beside the smoked salmon industry, the presence of persistent *L. monocytogenes* strains has also been reported in other food and processing environments in the vegetables, meat, dairy and seafood sectors, illustrating the ubiquitous nature of *L. monocytogenes* and the possible transfer of *L. monocytogenes* from the environment to finished food products [46, 47]. Due to the limited discriminatory power of the MLST technique, we could not rule out the possibility that Brand A’s salmon products were contaminated with variants of *L. monocy*togenes belonging to the same sequence types. However, this uncertainty could only be addressed by comparing the sequences of these strains using whole genome sequencing technique. The testing of raw salmon fish and environmental swabs of the manufacturing environment would also be required, to obtain isolates for comparison with those detected in the finished products, so as to confirm the presence and locations of persistence *L. monocytogenes* strains. Nevertheless, the high *L. monocytogenes* positivity rate in unsealed pre-packed smoked salmon at supermarkets highlighted a need to improve manufacturing hygiene processes. The observations of significantly higher prevalence of *L. monocytogenes* in smoked salmon from salad bars as compared to supermarkets, suggested that the bacteria could have proliferated along the food supply chain as a result of time-temperature abuse during distribution and storage, and/or cross-contamination during retail food preparation. We observed that the typical shelf life of pre-packed smoked salmon sold in supermarkets was about one to two months, which was a fairly extended period of time that could allow *L. monocytogenes* to proliferate to high levels. The corresponding higher SPC substantiates the need for improved manufacturing processes and cold chain management along the food distribution network.

Our study on pre-packed salad dishes and salad bar ingredients shows two interesting findings; the detection of *L. monocytogenes* ST155 in pre-packed chicken salads sampled from two geographically distinct outlets of a salad bar chain (Chain A), and the detection of *L. monocytogenes* ST87 in a smoked salmon sample and a chilled cooked shrimp sample from an outlet of a salad bar chain (Chain C). The detection of strains with common molecular profiles across and within retail facilities has been reported previously [41, 48] and this could be due to several reasons. For instance, these sequence types may be common in the environment which could thus explain their presence in multiple unrelated sources [48]. It may be because smoked salmon used by retailers came from a common supplier [48]. Within a food preparation environment, cross-contamination between food and the environment could also have occurred [41]. However, as the discriminatory power of molecular characterisation techniques such as MLST and pulsed-field gel electrophoresis are limited [48], the hypotheses mentioned could only be verified by conducting further studies to obtain information on the sources of smoked salmon used by retailers and compare the sequences of *L. monocytogenes* strains isolated from food and the environments at various manufacturing plants and retail establishments using whole genome sequencing techniques.

The most frequently encountered *L. monocytogenes* serotypes in pre-packed smoked salmon from supermarkets were 4b and 1/2b. Among the 13 known *L. monocytogenes* serotypes, 4b, 1/2b, 1/2a and 1/2c are the serotypes most commonly associated with human listeriosis [49]. A review of literature [50–55] and records of the *L. monocytogenes* MLST database by the Institut Pasteur [56] showed that ST 2, ST7, ST87, ST155 and ST193 which were detected in this study, were reported previously in various food types. For instance, ST2 was previously detected in smoked salmon, cheese and meat, ST7 in cantaloupe and whipping cream, ST87 in vegetables and meats, ST155 in vegetables, meat, fish, seafood and ice-cream, as well as ST193 in salmon roes [51, 53–56]. There were also past records on the detection of ST2, ST7, ST87 and ST155 in the food production environments [55, 56] and ST7 and ST155 in rodents [50, 56]. Four sequence types detected in this study were also found to be associated with human listeriosis in various countries. These included the isolation of ST2 from maternal-foetal infection cases, central nervous system infection cases and stillborn infants, ST7 in stillborn infants, as well as ST87 and ST155 in bacteraemia, meningitis and peritonitis cases [50, 51, 56]. Although ST2, ST7, ST87 and ST155 have been associated with invasive listeriosis in other countries [50, 51, 56], there has not been a notable increase in listeriosis cases reported in Singapore in recent years. This may be because listeriosis is not a legally notifiable disease and therefore the number of human cases could have been underreported. Worldwide, the annual incidence of listeriosis was between 0.1 and 11.3 per million populations; with an increasing trend of human infections reported in Europe between 2008 and 2014 [57, 58]. The absence of notable increase in human listerosis cases in Singapore could also be due to the limitation of MLST technique in discriminating virulent and avirulent strains within the same sequence type. This warrants further studies to assess the virulence potential and unique characteristics of these strains by performing whole genome analyses.

### Unsatisfactory SPC in high proportion of pre-packed salads and salad ingredients: an indication of poor microbial quality or an overly stringent limit?

Although the median SPC of pre-packed salads, vegetable ingredients and seafood ingredients at salad bars exceeded the local SPC limit for ready-to-eat food (<5 log CFU/g) [37], the data should be interpreted with caution. SPC is often used to estimate the microbial load in food and to provide indication on the overall hygienic quality of a food item [59]. If high SPC is observed in cooked food, it implies that the food could have been subjected to undercooking, unhygienic handling and/or prolonged storage. As some samples collected in this study consisted of raw vegetables and cold smoked fish, higher SPC was expected in these uncooked ingredients [60, 61]. Overall, the SPC of vegetables ingredients and cold smoked salmon samples in our study were comparable to other studies [62–66]. Studies elsewhere have shown that bacteria tend to bind more tightly to rough surface such as lettuce leaves and could internalise plant tissues, and therefore, were not easily removed by washing [67]. Considering the inherent nature of raw vegetables and cold smoked fish, some countries do not set a maximum SPC limit for salads [59, 61, 68, 69] and set a higher limit (<7 log CFU/g) for cold smoked fish [59, 68, 69]. A review of the local SPC limits for such ready-to-eat retail food types is thus recommended.

On the other hand, the relatively high proportions of pasta, rice and couscous ingredients, as well as poultry and egg ingredients exceeding the SPC limit were not hygienically acceptable as this suggested the presence of post-cooking contamination, possibly due to improper handling, prolonged storage, inadequate chilling or a combination of these. In contrast, lower SPC was observed in pre-packed salad dressing and dressing ingredients from salad bars, probably because salad dressing are generally acidic and the growth of most bacteria is hindered by low pH [70].

### The occasional detection of *E. coli, S. aureus, B. cereus* in salads and salad ingredients highlighted a need to improve retail hygiene processes

As *E. coli* is part of the commensal gut flora in warm blooded animals, its presence is often used as a hygiene indicator for faecal contamination [71]. As fresh produce could easily come into contact with soil or organic fertilisers at the farm level, it was not surprising to detect *E. coli* occasionally in raw vegetable salads in our study (2.3%, 1/44). However, the presence of *E. coli* in chilled cooked ingredients, such as poultry and eggs (6.3%, 2/32), as well as pasta, rice and couscous (5.9%, 2/34) was not hygienically acceptable. As *E. coli* can be inactivated easily by heat treatment, its presence suggested that the ingredients were either undercooked or exposed to post-cooking contamination [71].

Although *S. aureus* was occasionally detected in vegetable ingredients (6.1%, 2/33), as well as poultry and eggs ingredients (3.1%, 1/32) from salad bars, the organism was not detected in any of the pre-packed salads tested. As *S. aureus* can be part of humans’ skin flora [72], its presence in salad bar ingredients was not surprising as such ingredients are usually handled extensively during processes like slicing, dicing and mixing. As the *S. aureus* isolates obtained in this study were not found to possess any enterotoxin genes and the counts were much lower than the level that can produce sufficient enterotoxins to cause food poisoning (5 log CFU/g) [12], these ingredients were unlikely to cause food poisoning if consumed. Nevertheless, the presence of *S. aureus* in these ingredients suggested that there were occasional lapses in personnel hygiene.

*B. cereus*, a spore-forming bacterium commonly found in soil [12], was detected more frequently in pre-packed salads (12.6%, 18/143) than in salad bar ingredients (2.0%, 4/198). As pre-packed salads were usually kept for a longer period compared to salad bar ingredients due to transportation and display processes, prolonged storage could have provided an opportunity for *B. cereus* to multiply. *B. cereus* was also observed to affect various salad bar ingredients to a smaller extent, possibly because the ingredients were left exposed to the environment during display, inadequate chilling or due to the use of common utensils to handle multiple ingredients. Although several virulence genes were detected in several *B. cereus* strains isolated in this study, the counts were much lower than the levels (5 – 8 log CFU/g) that are indicative of potential human hazard due to preformed toxins [12].

The present study highlighted several areas for improving retail food hygiene processes. For instance, cross-contamination at the retail level can be minimised by reinforcing good glove and hand hygiene practices, as well as segregating utensils meant for handling raw and ready-to-eat food [73, 74]. Besides imparting knowledge to food handlers, it is also important to create a strong industrial food safety culture; this could be done through the inclusion of factors such as management’s commitment, food handlers’ sense of personal responsibility and improving risk communication in the holistic development of food safety management systems [75, 76]. However, as *Listeria* is ubiquitous in the environment [12], it might be difficult to eradicate its presence completely in certain food types. Thus, at risk populations such as pregnant women, the elderly, young children and the immunocompromised individuals should refrain from consuming food prone to *Listeria* contamination [77–80].

## Conclusion

Our findings highlighted a potential health risk associated with *L. monocytogenes* in seafood salad ingredients, in particular smoked salmon. While our microbial survey has certain limitations in its sampling design as discussed above, our findings do point to a need for improved manufacturing and retail hygiene processes. Public health risk can be further reduced by educating vulnerable populations to avoid the consumption of food prone to *Listeria* contamination*.* Additionally, and although the relatively high SPC of raw vegetables in this study should be interpreted with caution, thorough washing of salad vegetables should not be neglected to improve the overall hygiene quality of salads. Our findings provided preliminary data that could be useful to support the need for a more in-depth risk assessment study on specific pathogens in retail salad dishes.

## Additional files

Additional file 1 Table S1. Descriptions of ingredients in pre-packed salads. **Table S2.** Breakdown of salad bar ingredients collected. (DOCX 17 kb)
Additional file 2
**Appendix S1.** Evaluating the use of Universal Pre-enrichment Broth (UPB) as a sample suspension-and-enrichment medium in the testing of multiple foodborne bacteria. **Figure S1.** Comparing the use of Universal Pre-enrichment Broth (UPB) and Butterfield’s Buffer (BF) as a sample suspension medium for the detection of Standard Plate Count (SPC), *E. coli* count (EC), *S. aureus* count (SA) and *B. cereus* count (BC). (DOCX 37 kb)
Additional file 3 Table S3. Primers used for the detection of *Listeria* spp. and *L. monocytogenes*, as well as the characterisation of *E. coli, S. aureus, B. cereus* and *L. monocytogenes* (DOCX 28 kb)
Additional file 4 Figure S2. Standard Plate Count (SPC) of smoked salmon at salad bars and pre-packed smoked salmon at supermarkets. (DOCX 27 kb)

## Abbreviations

ESBL: Extended spectrum beta lactamase
mCCDA: modified Charcoal-Cefoperazone-Desoxycholate agar
MLST: Multi Locus Sequence Typing
RAPD: Random amplified polymorphic DNA
SPC: Standard Plate Count
ST: Sequence Type
TCBS: Thiosulfate-Citrate-Bile Salt-Sucrose agar
