# Supplementary material for: Microbial survey of ready-to-eat salad ingredients sold at retail reveals the occurrence and the persistence of Listeria monocytogenes Sequence Types 2 and 87 in pre-packed smoked salmon
Source: BMC Microbiol. 2017 Feb 28;17:46. doi: 10.1186/s12866-017-0956-z (PMC5331722; doi:10.1186/s12866-017-0956-z)
Supplement: Additional file 1 Table S1. — Descriptions of ingredients in pre-packed salads. Table S2. Breakdown of salad bar ingredients collected. (DOCX 17 kb) [file 12866_2017_956_MOESM1_ESM.docx]

## **Additional File 1:** Table S1, Table S2.

Table S1. Descriptions of ingredients in pre-packed salads

| **Types of pre-packed salads** | **Ingredients declared on salad packaging** |
| --- | --- |
| Vegetable salads (n=44) | Capsicums, carrots, corns, cucumbers, leafy greens (butterhead lettuce, cabbage, cavelo nero, celery, chards, coral lettuce, endives, flat leaf parsley, frisee, landcress, mustard greens, green frills, mizuna, oak leaf lettuce, radicchio, raisins, red veined sorrels, romaine lettuce, salad rockets, spinach, watercress), peas, potatoes, raisins, red onions, and/or tomatoes. |
| Chicken salads (n=32) | Cooked chicken and other ingredients such as avocado, button mushrooms, carrots, capsicum, celery, cheese, croutons, cucumbers, lettuce, onions, raisins, potatoes, thyme, tomatoes and/or turkey bacon bits. |
| Pasta, rice and couscous salads (n=30) | Cooked cereal based ingredients (pasta, rice and/or couscous) and other ingredients such as carrot, capsicum, celery, corn, dill, garlic, onion, olives, parsley, peas, potato, pumpkins, thyme, raisin, sausages and/or sesame seeds. |
| Pre-packed dressing (n=37) | Caesar dressing, mayonnaise, ranch dressing, thousand island and vinaigrette. |

Table S2. Breakdown of salad bar ingredients collected

| **Types of salad bar ingredients** | **Breakdown of samples collected** |
| --- | --- |
| Seafood (n=34) | Cold smoked salmon (n=15), cooked prawns/shrimps (n=8), tuna mayonnaise (n=6), raw tuna (n=5) |
| Dressing (n=34) | Vinaigrette (n=14), Caesar dressing (n=9), thousand island (n=4), ranch dressing (n=3), wasabi honey soy dressing (n=3), mayonnaise (n=1) |
| Pasta, rice and couscous (n=34) | Pasta (n=20), brown rice (n=8), couscous (n=6) |
| Vegetables (n=33) | Cucumbers (n=11), tomatoes (n=11), mixed leafy greens (information on the types of vegetables used were not provided) (n=11) |
| Poultry and eggs (n=32) | Cooked chicken (n=22), hard boiled eggs (n=7), egg mayonnaise (n=3) |
| Cheese (n=31) | Cheddar cheese (n=13), feta cheese (n=8), parmesan cheese (n=6), Swiss cheese (n=4) |
