# Supplementary material for: Microbial survey of ready-to-eat salad ingredients sold at retail reveals the occurrence and the persistence of Listeria monocytogenes Sequence Types 2 and 87 in pre-packed smoked salmon
Source: BMC Microbiol. 2017 Feb 28;17:46. doi: 10.1186/s12866-017-0956-z (PMC5331722; doi:10.1186/s12866-017-0956-z)
Supplement: Additional file 2 — Appendix S1. Evaluating the use of Universal Pre-enrichment Broth (UPB) as a sample suspension-and-enrichment medium in the testing of multiple foodborne bacteria. Figure S1. Comparing the use of Universal Pre-enrichment Broth (UPB) and Butterfield’s Buffer (BF) as a sample suspension medium for the detection of Standard Plate Count (SPC), E. coli count (EC), S. aureus count (SA) and B. cereus count (BC). (DOCX 37 kb) [file 12866_2017_956_MOESM2_ESM.docx]

## **Additional File 2:** Appendix S1, Figure S1.

## **Appendix S1: Evaluating the use of Universal Pre-enrichment Broth (UPB) as a sample suspension-and-enrichment medium in the testing of multiple foodborne bacteria**

Materials and methods

To improve laboratory efficiency, we evaluated the use of UPB as a sample suspension medium for determining SPC, *E. coli* count, *S. aureus* count and *B. cereus* count, as well as an enrichment medium for the recovery of *Salmonella* spp., *L.* *monocytogenes, V. cholerae*, *V. parahaemolyticus* and *E. coli* O157: H7. The workflow is described as follows.

1. Bacterial strains

Two bacterial concoctions, each consisting of seven reference organisms were used in the evaluation of UPB. The organisms in concoction 1 were *B. cereus* ATCC 11778, *E. coli* ATCC 25922, *E. coli* O157: H7 (ATCC 43888), *L. monocyotogenes* ATCC 7644, *S.* Typhimurium ATCC 19585, *S. aureus* ATCC 25923 and *V. parahaemolyticus* ATCC 17802. Concoction 2 included *B. cereus* ATCC 27877, *E. coli* ATCC 43893, *E. coli* O157:H7 ATCC 43888, *L. monocyotogenes* FSL C1-56, *S. aureus* ATCC 19095, *S.* Typhimurium ATCC 13076 and *V. cholerae* AS5/96.

All reference strains were purchased from the American Type Culture Collection (ATCC), except for *L. monocytogenes* FSL C1-56 which was from the Cornell University, New York and *V. cholerae* AS5/96 from DSO National Laboratories, Singapore.

1. Preparation of inocula

Fresh inocula of each reference strain, except for *Vibrio* spp., were prepared by streaking a 10 µl-loopful of the frozen glycerol stock onto Tryptic Soy Agar (TSA) (Acumedia, Michigan) and incubated at 37^o^C for 18 hours. Reference *Vibrio* strains were grown on TSA with 2% sodium chloride (Promega, Madison) and incubated under the same condition. Pure colonies from each reference culture were suspended in 0.85% physiological saline to obtain an optical density of 0.1 at 595 nm (Ultrospec10 cell density meter, Amersham Biosciences), except for *B. cereus* and *Vibrio* spp. where an optical density of 0.4 was needed. This allowed a suspension of approximately 10^7^CFU/ml to be obtained for each bacterial strain. The exact concentrations of each bacterial suspension were verified by plating onto TSA plates containing 2% sodium chloride (for *Vibrio* strains) and TSA plates (for other bacteria strains) after appropriate serial dilutions (neat to 10^8^ dilutions).

1. Determination of SPC, *E. coli*, *S. aureus*, and *B. cereus* counts from pre-enriched UPB and Butterfield’s buffer

One millilitre of the 100x dilution of each reference strain in Concoction 1 was spiked into 100 ml of UPB and 100 ml of Butterfield’s buffer (3M, Minnesota) respectively and homogenised to obtain a suspension containing approximately 10^3^ CFU/ml of each reference strain. Fifteen minutes after spiking, the suspension at ambient condition was subjected to the analysis of SPC, *E. coli* count, *S. aureus* count and *B. cereus* count using the methods described in the section on microbiological analyses. The experiment was repeated with reference strains in concoction 2. Eight replicates of UPB spiked with each concoction were prepared.

1. Recovery of *E. coli* O157: H7, *L. monocytogenes, Salmonella* spp. and *Vibrio* spp., from enriched Universal Pre-enrichment Broth (UPB)

The spiked UPB suspensions were incubated at 37^o^C for 18 hours. The recovery of *E. coli* O157: H7, *L. monocytogenes*, *Salmonella* spp. and *Vibrio* spp. were performed using the methods described in the section on microbiological analyses.

Results

No significant differences in Standard Plate Count (SPC), *E. coli* count, *S. aureus* count and *B. cereus* count were observed (see Supplementary Figure S1) when UPB and Butterfield’s buffer were used as sample suspension media. *E. coli* O157: H7, *L. monocytogenes, Salmonella* spp. and *Vibrio* spp. were recovered from all replicates of enriched UPB spiked with the two respective concoctions.


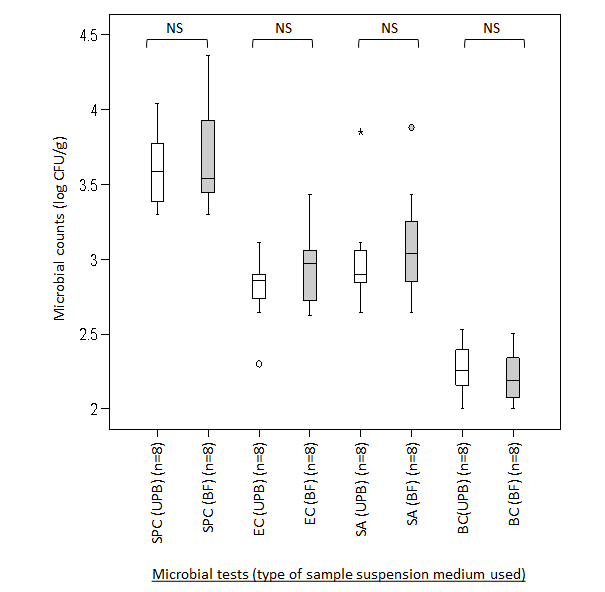


**Figure S1: Comparing the use of Universal Pre-enrichment Broth (UPB) and Butterfield’s Buffer (BF) as a sample suspension medium for the detection of Standard Plate Count (SPC), *E. coli* count (EC), *S. aureus* count (SA) and *B. cereus* count (BC)**

NS: The difference in bacterial count was not significant (p>0.05)

^o,*^: Outliers
