# Supplementary material for: Microbial survey of ready-to-eat salad ingredients sold at retail reveals the occurrence and the persistence of Listeria monocytogenes Sequence Types 2 and 87 in pre-packed smoked salmon
Source: BMC Microbiol. 2017 Feb 28;17:46. doi: 10.1186/s12866-017-0956-z (PMC5331722; doi:10.1186/s12866-017-0956-z)
Supplement: Additional file 3 Table S3. — Primers used for the detection of Listeria spp. and L. monocytogenes, as well as the characterisation of E. coli, S. aureus, B. cereus and L. monocytogenes (DOCX 28 kb) [file 12866_2017_956_MOESM3_ESM.docx]

**Additional File 3:** Table S3

| Table S3. Primers used for the detection of *Listeria* spp. and *L. monocytogenes,* as well as the characterisation of *E. coli, S. aureus, B. cereus* and *L. monocytogenes* | | | | | | | |  |
| --- | --- | --- | --- | --- | --- | --- | --- | --- |
| **Parameter** | **Target gene** | **Forward primer (5'-3')** | **Reverse primers (5'-3')** | **Concentration (µM)** | **Size**  **(bp)** | **References** |  |  |
| Enteropathogenic *E. coli* (EPEC) | *eaeA* | TCAATGCAGTTCCGTTATCAGTT | GTAAAGTCCGTTACCCCAACCTG | 0.05µM | 482 | [19-21] |  |  |
|  | *escV* | ATTCTGGCTCTCTTCTTCTTTATGGCTG | CGTCCCCTTTTACAAACTTCATCGC | 0.05µM | 544 |  |  |  |
|  | *ent* | TGGGCTAAAAGAAGACACACTG | CAAGCATCCTGATTATCTCACC | 0.05µM | 629 |  |  |  |
|  | *bfpβ* | GACACCTCATTGCTGAAGTCG | CCAGAACACCTCCGTTATGC | 0.05µM | 910 |  |  |  |
| Enteroaggregative *E. coli* (EAEC) | *aggR* | ACGCAGAGTTGCCTGATAAAG | AATACAGAATCGTCAGCATCAGC | 0.2µM | 400 |  |  |  |
|  | *pic* | AGCCGTTTCCGCAGAAGCC | AAATGTCAGTGAACCGACGATTGG | 0.05µM | 1111 |  |  |  |
|  | *astA* | TGCCATCAACACAGTATATCCG | ACGGCTTTGTAGTCCTTCCAT | 0.2µM | 102 |  |  |  |
| Shiga toxin-producing *E. coli* (STEC) | *eaeA* | TCAATGCAGTTCCGTTATCAGTT | GTAAAGTCCGTTACCCCAACCTG | 0.05µM | 482 |  |  |  |
|  | *ehxA* | GGTGCAGCAGAAAAAGTTGTAG | TCTCGCCTGATAGTGTTTGGTA | 0.15µM | 1551 |  |  |  |
|  | *stx1* | CGATGTTACGGTTTGTTACTGTGACAGC | AATGCCACGCTTCCCAGAATTG | 0.1µM | 244 |  |  |  |
|  | *stx2* | GTTTTGACCATCTTCGTCTGATTATTGAG | AGCGTAAGGCTTCTGCTGTGAC | 0.1µM | 324 |  |  |  |
| Enterotoxigenic *E. coli* (ETEC) | *estlα* | CCTCTTTTAGYCAGACARCTGAATCASTTG | CAGGCAGGATTACAACAAAGTTCACAG | 0.05µM | 157 |  |  |  |
|  | *estlβ* | TGTCTTTTTCACCTTTCGCTC | CGGTACAAGCAGGATTACAACAC | 0.05µM | 171 |  |  |  |
|  | *elt* | GAACAGGAGGTTTCTGCGTTAGGTG | CTTTCAATGGCTTTTTTTTGGGAGTC | 0.05µM | 655 |  |  |  |
| Methicillin-resistant  *S. aureus* | *mecA* | AAAATCGATGGTAAAGGTTGGC | AGTTCTGCAGTACCGGATTTGC | 0.2μM | 533 | [22-24] |  |  |
| *S. aureus* enterotoxin-A genes | *SEA* | ACGATCAATTTTTACAGC | TGCATGTTTTCAGAGTTAATC | 0.2μM | 544 |  |  |  |
|  |  |  |  |  |  |  |  |  |
| *S. aureus* enterotoxin-B genes | *SEB* | GAATGATATTAATTCGCATC | TCTTTGTCGTAAGATAAACTTC | 0.2μM | 416 |  |  |  |
| *S. aureus* enterotoxin-C genes | *SEC* | GACATAAAAGCTAGGAATTT | AAATCGGATTAACATTATCCA | 0.2μM | 257 |  |  |  |
| *S. aureus* enterotoxin-D genes | *SED* | TTACTAGTTTGGTAATATCTCCTT | CCACCATAACAATTAATGC | 0.2μM | 334 |  |  |  |
| *S. aureus* enterotoxin-E genes | *SEE* | ATAGATAAAGTTAAAACAAGCAA | TAACTTACCGTGGACCC | 0.2μM | 170 |  |  |  |
| *S. aureus* enterotoxin-G genes | *SEG* | ACGTCTCCACCTGTTGAAGG | TGAGCCAGTGTCTTGCTTTG | 0.1μM | 400 |  |  |  |
| *S. aureus* enterotoxin-H genes | *SEH* | TCACATCATATGCGAAAGCAG | TAGCACCAATCACCCTTTCC | 0.1μM | 357 |  |  |  |
| *S. aureus* enterotoxin-I genes | *SEI* | TGGAACAGGACAAGCTGAAA | TAAAGTGGCCCCTCCATACA | 0.2μM | 426 |  |  |  |
| *S. aureus* enterotoxin-J genes | *SEJ* | CAGCGATAGCAAAAATGAAACA | TCTAGCGGAACAACAGTTCTGA | 0.2μM | 467 |  |  |  |
| *S. aureus* enterotoxin-L genes | *SEL* | CACCAGAATCACACCGCTTA | CTGTTTGATGCTTGCCATTG | 0.2μM | 240 |  |  |  |
| Emetic toxin gene of *B. cereus* | *cer* | AACGTCGGTATGATTTTAGG | CTCTTCTGCTCTCTATTTATGTC | 0.05μM | 234 | [25-32] |  |  |
| Diarrhoeal toxin gene of *B. cereus* | *hblC* | AATAGGTACAGATGGAACAGG | GGCTTTCATCAGGTCATACTC | 0.05μM | 399 |  |  |  |
|  | *hblD* | AATCAAGAGCTGTCACGAAT | CACCAATTGACCATGCTAAT | 0.05μM | 439 |  |  |  |
|  | *hblA* | GCTAATGTAGTTTCACCTGTAGCAAC | AATCATGCCACTGCGTGGACATATAA | 0.1μM | 883 |  |  |  |
|  | *nheA* | TACGCTAAGGAGGGGCA | GTTTTTATTGCTTCATCGGCT | 0.05μM | 500 |  |  |  |
|  | *nheB* | CTATCAGCACTTATGGCAG | ACTCCTAGCGGTGTTCC | 0.05μM | 770 |  |  |  |
|  | *nheC* | CGGTAGTGATTTGCTGGG | CAGCATTCGTACTTGCCAA | 0.05μM | 582 |  |  |  |
|  | *cytK* | ACAGATATCGGKCAAAATGC | TCCAACCCAGTTWSCAGTTC | 0.05μM | 809 |  |  |  |
|  | *entFM* | ATGAAAAAAGTAATTTGCAGG | TTAGTATGCTTTTGTGTAACC | 0.15μM | 1300 |  |  |  |
|  | *bceT* | TTACATTACCAGGACGTGCTT | TGTTTGTGATTGTAATTCAGG | 0.05μM | 428 |  |  |  |
| Haemolysin II gene of *B. cereus* | *hlyll* | CAAGTTACTCTTGATAACC | TCACCATTTACAAAGATACC | 0.2μM | 194 |  |  |  |
|  |  |  |  |  |  |  |  |  |
| *Listeria* spp. | *prs* | GCTGAAGAGATTGCGAAAGAAG | CAAAGAAACCTTGGATTTGCG | 0.1μM | 370 | Modified [33] |  |  |
| *Listeria monocytogenes* | *inlA* | ACGAGTAACGGGACAAATGC | CCCGACAGTGGTGCTAGATT | 0.1μM | 800 | [34] |  |  |
|  |  |  |  |  |  |  |  |  |
|  |  |  |  |  |  |  |  |  |
| *L. monocytogenes* MLST | *abcZ* | GTTTTCCCAGTCACGACGTTGTATCGCTGCTGCCACTTTTATCCA | TTGTGAGCGGATAACAATTTCTCAAGGTCGCCGTTTAGAG | 0.1μM | 537 |  |  |  |
|  | *bglA* | GTTTTCCCAGTCACGACGTTGTAGCCGACTTTTTATGGGGTGGAG | TTGTGAGCGGATAACAATTTCCGATTAAATACGGTGCGGACATA | 0.1μM | 399 |  |  |  |
|  | *cat* | GTTTTCCCAGTCACGACGTTGTAATTGGCGCATTTTGATAGAGA | TTGTGAGCGGATAACAATTTCAGATTGACGATTCCTGCTTTTG | 0.06μM | 486 | [35] |  |  |
|  | *dapE* | GTTTTCCCAGTCACGACGTTGTACGACTAATGGGCATGAAGAACAAG | TTGTGAGCGGATAACAATTTCATCGAACTATGGGCATTTTTACC | 0.1μM | 462 |  |  |  |
|  | *dat* | GTTTTCCCAGTCACGACGTTGTAGAAAGAGAAGATGCCACAGTTGA | TTGTGAGCGGATAACAATTTCTGCGTCCATAATACACCATCTTT | 0.06μM | 471 |  |  |  |
|  | *ldh* | GTTTTCCCAGTCACGACGTTGTAGTATGATTGACATAGATAAAGA | TTGTGAGCGGATAACAATTTCTATAAATGTCGTTCATACCAT | 0.1μM | 453 |  |  |  |
|  | *lhkA* | GTTTTCCCAGTCACGACGTTGTAAGAATGCCAACGACGAAACC | TTGTGAGCGGATAACAATTTCTGGGAAACATCAGCAATAAAC | 0.1μM | 480 |  |  |  |
|  |  |  |  |  |  |  |  |  |
|  |  |  |  |  |  |  |  |  |
|  |  |  |  |  |  |  |  |  |
|  |  |  |  |  |  |  |  |  |
