# Supplementary material for: Microbial survey of ready-to-eat salad ingredients sold at retail reveals the occurrence and the persistence of Listeria monocytogenes Sequence Types 2 and 87 in pre-packed smoked salmon
Source: BMC Microbiol. 2017 Feb 28;17:46. doi: 10.1186/s12866-017-0956-z (PMC5331722; doi:10.1186/s12866-017-0956-z)
Supplement: Additional file 4 Figure S2. — Standard Plate Count (SPC) of smoked salmon at salad bars and pre-packed smoked salmon at supermarkets. (DOCX 27 kb) [file 12866_2017_956_MOESM4_ESM.docx]

**Additional File 4:** Figure S2


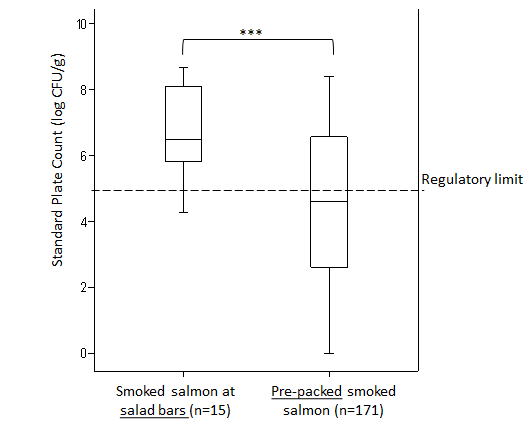


**Figure S2. Standard Plate Count (SPC) of smoked salmon at salad bars and pre-packed smoked salmon at supermarkets**

***: The difference in SPC was significant (p<0.001)

---: Singapore’s regulatory limit for SPC in ready-to-eat food (<5 log CFU/g) [37]
